# A Systematic Review of Studies Published between 2016 and 2019 on the Effectiveness and Efficacy of Pneumococcal Vaccination on Pneumonia and Invasive Pneumococcal Disease in an Elderly Population

**DOI:** 10.3390/pathogens9040259

**Published:** 2020-04-03

**Authors:** Jacob Dag Berild, Brita Askeland Winje, Didrik Frimann Vestrheim, Hans-Christian Slotved, Palle Valentiner-Branth, Adam Roth, Jann Storsäter

**Affiliations:** 1 Department of Vaccine Preventable Diseases, Division of Infection Control and Environmental Health, Norwegian Institute of Public Health, 0213 Oslo, Norway; Brita.Askeland.Winje@fhi.no (B.A.W.); DidrikFrimann.Vestrheim@fhi.no (D.F.V.); 2 Department of Bacteria, Parasites and Fungi, Statens Serum Institute, 2300 Copenhagen, Denmark; hcs@ssi.dk; 3 Infectious Disease Epidemiology & Prevention, Statens Serum Institute, 2300 Copenhagen, Denmark; pvb@ssi.dk; 4 Unit for Vaccination Programmes, Public Health Agency of Sweden, 171 82 Solna, Sweden; adam.roth@folkhalsomyndigheten.se (A.R.); jann.storsater@folkhalsomyndigheten.se (J.S.)

**Keywords:** pneumococcal vaccines, review, invasive pneumococcal disease, pneumonia, elderly

## Abstract

Adult vaccination is high on the agenda in many countries. Two different vaccines are available for the prevention of pneumococcal disease in adults: a 23-valent polysaccharide vaccine (PPV23), and a 13-valent conjugated vaccine (PCV13). The objective of this review is to update the evidence base for vaccine efficacy and effectiveness of PPV23 and PCV13 against invasive pneumococcal disease and pneumonia among an unselected elderly population. We systematically searched for clinical trials and observational studies published between January 1 2016 and April 17 2019 in Pubmed, Embase, Cinahl, Web of Science, Epistemonikos and Cochrane databases. Risk of bias was assessed using Cochrane Risk of Bias tool for and the Newcastle–Ottawa Scale. Results were stratified by vaccine type and outcome. We identified nine studies on PCV13 and six on PPV23. No new randomized clinical trials were identified. Due to different outcomes, it was not possible to do a meta-analysis. New high-quality observational studies indicate protective vaccine effectiveness for both vaccines against vaccine type pneumonia. Our estimates for the protective vaccine efficacy and effectiveness (VE) of PPV23 on pneumonia and pneumococcal pneumonia overlap with results from previously published reviews. Some of the results indicate that the effectiveness of the PPV23 is best in younger age groups, and that it decreases over time.

## 1. Introduction

*Streptococcus pneumoniae* is a leading cause of mortality and morbidity in the elderly population. The most severe form of pneumococcal disease is invasive pneumococcal disease (IPD). Additionally, *S. pneumoniae* can cause non-invasive disease such as pneumonia, sinusitis and otitis media. Estimates show that the number of hospitalizations due to pneumococcal pneumonia will double in the US from 2004 to 2040 without any interventions, and that this will lead to a $ 2.5 billion increase in health care expenditure [1]. In an effort to reduce this burden, two different vaccines have been developed - a 23-valent polysaccharide vaccine (PPV23, Pneumovax 23, Sanofi Pasteur, MSD) and a 13-valent conjugate vaccine (PCV13, Prevenar13, Pfizer). With the exception of serotype 6A, all serotypes in PCV13 are also included in PPV23.

The randomized placebo-controlled trial named CAPITA demonstrated the efficacy of PCV13 in adults aged ≥ 65 years. The study was conducted in the Netherlands, included almost 85,000 participants, and found a modified intention-to-treat vaccine efficacy of 37.7% (95% CI: 14.3 to 55.1) against the first episode of vaccine-type community acquired pneumococcal pneumonia (VT-CAP) and 75.8% (95% CI: 46.5 to 90.3) against the first episode of vaccine-type invasive pneumococcal disease (VT-IPD) [2].

In 2016 and 2017, five different systematic reviews evaluated the vaccine efficacy and effectiveness (hereafter both will be abbreviated VE) of the PPV23 vaccine [3,4,5,6,7] (Table S1). The pooled VE on invasive pneumococcal disease was estimated as 73% in randomized controlled trials (RCTs) [4] and as 45–59% in observational studies [4,5] (Table S1). However, the estimated collected body of evidence on pneumococcal pneumonia varied greatly between the reviews—some reviews indicated a statistically significant VE [4,5,7], while others did not [3,6] (Table S1). The disparate results reflect different inclusion and exclusion criteria for studies and designs. Many of the reviews also reported on all-cause pneumonia [3,5,6,7], but none of the systematic reviews reported on vaccine-type (VT) pneumonia. Against this background the Norwegian Institute of Public Health, Statens Serum Institute in Denmark and the Public Health Agency of Sweden, we conducted a systematic review to update the evidence base for VE of pneumococcal vaccines against invasive pneumococcal disease and pneumonia in a general elderly population. 

## 2. Materials and Methods 

### 2.1. Literature Search

We systematically searched for eligible publications in Pubmed, Embase, Cinahl, Web of Science, Epistemonikos and Cochrane databases. 

To avoid repeating previous work [3,4,5,6,7], we only considered publications from January 1 2016 and onwards. 

Eligible studies were original reports on VE of the PCV13 or PPV23 in a general elderly population. Thus, we excluded studies that only included high-risk patients. We did not choose a specific age cut-off in order to avoid missing any important publications. The comparator was no vaccine or a placebo vaccine. We only considered studies reporting pneumonia and/or IPD as clinical outcomes, including all-cause, pneumococcal and/or serotype-specific disease. We did not set an a priori case definition for pneumonia or IPD, but used the case definitions used by respective authors. Both observational studies and clinical trials were included. Publication language was restricted to English, German, French, Spanish, Dutch, Danish, Norwegian or Swedish language. Furthermore, we excluded case reports, animal, modelling, immunogenicity, and carriage studies, health economic evaluations, and studies on the indirect VE of pneumococcal vaccination. Appendix A contains the complete electronic search strategy.

We conducted the search August 23 2018 with the support from a scientific librarian. Two reviewers (J.D.B, J.S.) independently extracted eligible studies according to the PICO (Population, Intervention, Comparator and Outcome) (Appendix C). We first screened by title and abstract, then by reading the full text. Any disagreement was resolved in consensus, and if necessary, with the help of a third reviewer (B.A.W.). We repeated this process on April 17 2019, to check for new publications. 

### 2.2. Data Extraction

J.D.B and B.A.W. extracted data from eligible studies using a predefined form (Appendix B). The main outcomes were VE on pneumonia and/or IPD, including all-cause pneumococcal and serotype-specific disease. Results were stratified by study design, vaccine type, outcome and age.

### 2.3. Quality Assessment

We assessed the quality of randomized trials using Cochrane Collaboration’s tool for assessing risk of bias [8], and observational studies using the Newcastle–Ottawa Scale (NOS) [9,10]. 

### 2.4. Analysis

VE estimates from the studies are presented individually. 

## 3. Results

**Figure 1:**
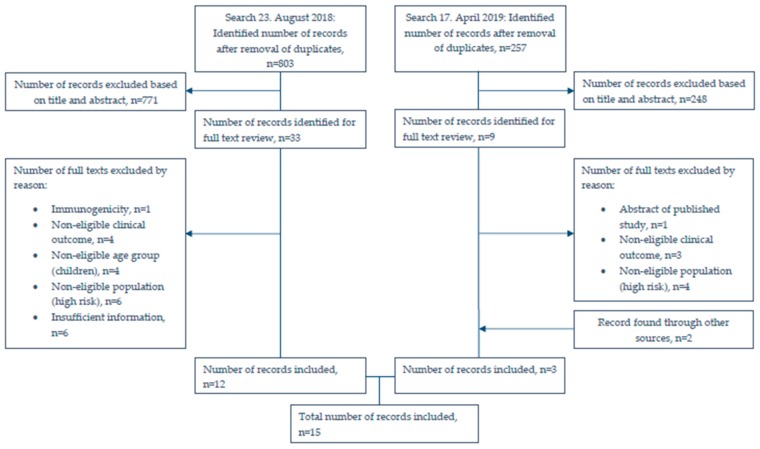
Flowchart of literature search. n: number.

After the removal of duplicates we identified 803 records, of which 771 were excluded based on title and abstract (Figure 1). After full text review, we included 12 studies (8 on PCV13 and 4 on PPV23). The updated search conducted April 17 2019 yielded 257 new records, from which one new PCV13 study was included. We also included two additional PPV23 studies found through other sources. Table 1 provides an overview of the included studies.

### 3.1. Characteristics 

#### 3.1.1. Conjugate Vaccine

Nine studies reported on PCV13 VE [11,12,13,14,15,16,17,18,19] (Table 1). Five of these were post-hoc analyses based on CAPITA data [11,12,13,14,15]. Besides the post-hoc studies, we identified two cohort studies, one from Spain [16] and one from Germany [17], and two studies using test-negative design (TND), one from the US [18] and one from Italy [19]. The Spanish cohort study used routine data collected in 2015 to identify all-cause pneumonia and pneumococcal pneumonia cases. Cohort members were observed for almost 2,000,000 person-years. However, only about 7000 of these were in PCV13-vaccinated persons [16]. 

The German cohort study used routine insurance data to identify 11,395 people vaccinated with PCV13, of a cohort of more than 500,000 inhabitants living in Saxony between 2014 and 2016 [17]. The American TND study included 2034 CAP hospitalizations and identified 68 VT-CAP cases using a serotype-specific urinary antigen test developed by Pfizer. The remaining non VT-CAP patients were controls [18]. The Italian TND study identified 1867 eligible CAP patients between 2013 and 2015, but due to a low participation rate, they only included 182 CAP patients in their VE analysis. Using a range of different diagnostic tools, they identified 59 pneumococcal CAP cases, of which 39 were VT-CAP [19].

#### 3.1.2. Polysaccharide Vaccine

For PPV23 we identified one cohort study from Germany [20], one TND study from Japan [21], one indirect cohort study from United Kingdom (UK) [22] and three case–control studies from Spain [23], Japan [24] and South Korea [25], respectively (Table 1). The German cohort study included almost 739,000 inhabitants aged ≥60 years, retrospectively, using routine insurance data collected between 2010 and 2011. Almost 30% of these were vaccinated with PPV23 [20]. The Japanese TND study enrolled and analyzed 2036 patients hospitalized with pneumonia between 2001 and 2014. Of the 2036 patients, 419 were categorized as having pneumococcal pneumonia. Sputum analysis identified the majority of these cases [21]. The study from UK included 9847 IPD cases from the enhanced national surveillance of IPD in England and Wales. Among these, 6245 (63%) had available vaccination information, and were included in VE analysis using the indirect cohort (Broome) method, with 4423 VT IPD cases and 1822 non VT-IPD controls [22]. The case–control study from Spain recruited 1895 CAP cases and an equal amount of controls from 20 different hospitals between 2013 and 2015 [23]. The Japanese case–control study matched 234 outpatients treated for pneumonia between 2009 and 2014 with 438 controls without pneumonia [24]. In South Korea 148 IPD and 557 non-bacteremic pneumococcal pneumonias (NBPP) patients were matched to 295 and 557 hospital controls, respectively. The Korean study identified NBPP cases using sputum culture or urinary antigen test [25]. 

### 3.2. Reported Outcomes

#### 3.2.1. Conjugate Vaccine

Four of the five post hoc studies using CAPITA data provided VE estimates for PCV13 stratified by comorbidities [11,12,13] and exploratory outcomes [14]. The last post hoc study analyzed the VE of time since vaccination and did not find evidence of waning over the study period [15]. The remaining studies reported VE on the following outcomes: all-cause pneumonia [16,17], pneumococcal pneumonia [16], pneumococcal-CAP [19] and VT-CAP [18,19] (Table 1). None of the observational studies reported on IPD. 

For the main outcomes, pneumonia and/or IPD, none of the CAPITA post hoc studies provided any significant new data compared to the original study [2], Hence, we did not quality assess them or extract any VE data on pneumonia or IPD (Table 1). 

#### 3.2.2. Polysaccharide Vaccine

VE of the PPV23 vaccine was reported on the following outcomes: all-cause pneumonia [20], CAP [23,24], non-bacteremic pneumococcal pneumonia (NBPP) [25], VT-NBPP [25], pneumococcal pneumonia [21] and VT-pneumonia [21], IPD [25] and VT-IPD [22,25] (Table 1).

### 3.3. Risk of Bias

We judged the Italian TND study on PCV13 [19], the German cohort on PPV23 [20], the Spanish case–control on PPV23 [23] and the Japanese case–control study on PPV23 [24] to be of low quality according to NOS criteria (Table 1 + Table S2). The Italian study had a low inclusion rate of only 10%, stopped before planned time, and did not adjust for confounding factors [19]. The German cohort study did not adequately describe the follow up of the cohort, and the follow up time was only two years [20]. The Spanish case–control used medical records to judge vaccination status. The included cases and controls had a lower vaccination coverage than average in this region and 90% had one or more comorbidity; this suggests some selection and information bias [23]. In the Japanese case–control study vaccination status and comorbidities were based on self-reporting, introducing a risk of recall bias. Furthermore, there was no information about non-response rate [24]. 

We judged the remaining observational studies to be of high quality according to the NOS (Table 1 + Table S2). Although we categorized the Spanish cohort study evaluating PCV13 VE as high quality according to the NOS, we found some important problems. The vaccinated cohort was significantly sicker and older, with 34% having an immunocompromising condition and 27% were 80 years or older. The follow-up period was only one year, and the vaccination rate was only 0.2% [16]. 

### 3.4. Vaccine efficacy/Effectiveness

#### 3.4.1. Conjugate Vaccine

**Figure 2:**
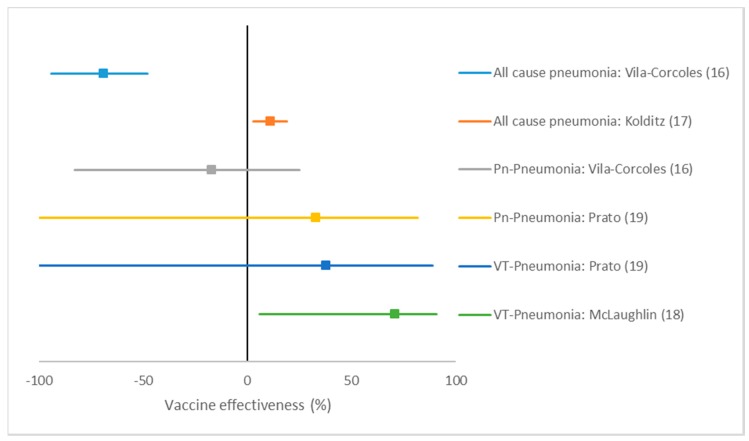
Forest plot of PCV 13 vaccine effectiveness estimates without summary estimate. Pn: pneumococcal. VT: Vaccine-type.

The Spanish cohort study did not find any protective VE on all-cause pneumonia or pneumococcal pneumonia (Table 2 + Figure 2). The German cohort study found an adjusted VE of 11% (95% CI: 3 to 19) against all-cause pneumonia. The American TND study found an adjusted VE of 71% (95% CI: 6 to 91) against VT-CAP. The Italian TND study found a crude VE of 33% (95% CI: -107 to 82) against pneumococcal CAP and a crude VE of 38% (95% CI: -132 to 89) against VT-CAP. 

#### 3.4.2. Polysaccharide Vaccine

**Figure 3:**
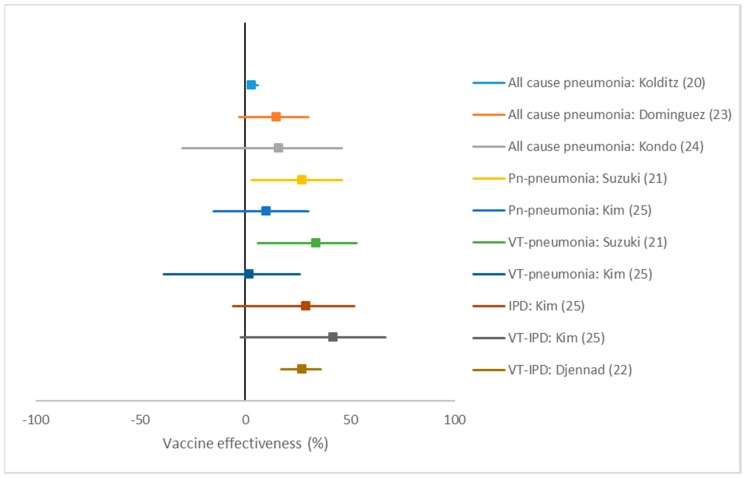
Forest plot of PPV23 vaccine effectiveness estimates without summary estimate. Pn: pneumococcal. VT: Vaccine-type. IPD: invasive pneumococcal disease.

The German cohort study did not present any relative measure in their results section, but using their numbers we calculated an adjusted (propensity score matched) VE of 3% (95% CI: 1 to 6) against all-cause pneumonia (Table 2 + Figure 3). The Japanese TND study found an adjusted VE of 27% (95% CI: 3 to 46) against pneumococcal pneumonia and a VE of 34% (95% CI: 6 to 53) against VT-pneumonia. The Spanish case–control study found an adjusted VE of 15% (95% CI: −3 to 30) against CAP. The Japanese case–control study found a similar adjusted VE of 16% (95% CI: −30 to 46) against CAP. The South Korean case–control study found an adjusted VE of 10% (95% CI: −15 to 30) against NBPP and 29% (95% CI: −6 to 52) against IPD. For VT-NBPP and VT-IPD the VE was −2% (95% CI: −40 to 26) and 42% (95% CI: −2 to 67), respectively. The UK study reported a VE of 27% (95% CI: 17 to 35) against VT-IPD.

### 3.5. Age

Five studies on PPV23 [20,21,22,23,25] reported VE stratified by age groups (Table 3). For PPV23 there seems to be a decreasing VE with increasing age, and only one study [22] reported a significant VE in the oldest age group (≥85 years). For PCV13, one study showed decreasing VE by age results [16], while another did not find any difference [17] (Table 3). 

## 4. Discussion

### 4.1. General Comments

Studies evaluating the VE of pneumococcal vaccines for the prevention of pneumonia are difficult to compare due to a lack of a standardized outcome definitions and diagnostic tools. The outcomes in the included studies in our systematic search ranged from all-cause pneumonia to VT-IPD. The cohort studies used routine data based on International Classification of Disease codes, while all other studies validated their cases using comparable clinical criteria (i.e., radiological findings combined with clinical symptoms and paraclinical observations). Most studies combined bacteremic and non-bacteremic pneumonias, and only one study analyzed non-bacteremic pneumonias. 

The usual way to identify pneumococcal bacteremia is by blood culture; however, this is not a feasible way to identify non-bacteremic pneumococcal pneumonias [26]. The studies in our review used several different tests to identify pneumococcal pneumonias; blood culture, sputum culture, sputum PCR, and urinary antigen tests, either as the BinaxNOW, a non serotype-specific test, or as the serotype-specific SSUAD developed to identify PCV13 serotypes. Some studies used a wide range of tests, while others used one or two. 

Other factors that can explain the large differences in VE are serotype distribution, background pneumococcal vaccination coverage, different study designs, characteristics of included participants, time since vaccination, and follow-up time. 

The main limitation of this review is the short time span of the systematic search. The low number of new publications did not permit meta-analyses stratified by vaccine, study design and outcome. However, our results can be used in conjunction with earlier studies not included in this review [2,3,4,5,6,7] (Table S1). Another limitation is the inconsistent use of age groups across studies, making it difficult to compare VE by age.

### 4.2. Evidence Base for PCV13

The VE in observational studies on PCV13 ranges from a negative VE (adjusted VE −69%) against all-cause pneumonia to a high protective VE of 71% against VT-CAP. All of these studies were conducted in the period 2014 to 2016, and in regions where PCV13 is a part of the routine childhood immunization schedule. 

The post hoc studies using CAPITA data provided some interesting information. Huijts et al. retrospectively obtained information on comorbidities from medical records (from GPs, hospitals) for participants who were identified with vaccine-type CAP (n=139), and from ICPC codes from general practitioners for 40 427 CAPITA participants. The number of individuals with comorbidities was higher when data were obtained from medical records rather than from self-report [12]. This could suggest that the population in CAPITA actually was less healthy than previously reported. Patterson et al. studied the VE of time since vaccination, and did not find any evidence of waning over the study period of four years [15]. 

The TND study from the US had a surprisingly high VE against VT-CAP [18]. However, the wide confidence intervals (reflecting a relatively low amount of VT-CAP cases) does overlap with results from CAPITA. Eight percent of the VT-CAP cases were bacteremic, which is a higher proportion than in the other two TND-studies (where the proportion was one percent). When restricted to non-bacteremic VT-CAP, the VE was a bit lower and became statistically non-significant 68% (95% CI: −6 to 90). The pneumococcal vaccines have consistently been found to provide better protection against invasive than non-invasive disease, and if the proportion of bacteremic pneumonia is high compared to non-bacteremic pneumonia one would expect a higher VE. Furthermore, the median time since vaccination was only 157 days. This short time since vaccination might also contribute to the high observed VE. 

The Italian TND study mixed different diagnostic tools, included only 10% of eligible patients, stopped before planned time, and did not adjust for underlying factors [19]. This has probably introduced detection and selection bias. However, the calculated VE is similar to the VE found in CAPITA and overlaps with the VE from the well-conducted TND from the US. The TND design also has several methodological advantages as it can reduce health care seeking bias and misclassification bias [27]. The VE against pneumococcal pneumonia is relatively high (33%) and almost the same as against VT pneumonia (38%). In the study, PCV13 serotypes caused 66% of the pneumococcal pneumonias. Although this might seem unlikely in a region with PCV13 childhood vaccination, an IPD surveillance study from Italy between 2008 and 2014 only showed a small, and not statistically significant, reduction in PCV13 serotypes for those aged < 64 years [28].

In the large Spanish cohort study, only 0.2% were PCV vaccinated [16]. The authors found no protective VE of the vaccine. Actually, they found an increased risk of pneumonia after adjustment in the vaccinated group. However, the vaccinated group was significantly older, with more comorbidities, and a higher uptake of the polysaccharide and influenza vaccine, and this could have introduced selection bias. The PPV23 coverage amongst elderly in this region of Spain have been high for many years. This could also lead to a lower estimated VE of the conjugate vaccine in Spain compared to CAPITA results from the Netherlands with a very low background PPV23 vaccination rate in the elderly population. Finally, this region of Spain has had a large reduction in circulating PCV13 serotypes due to the indirect effect of the PCV childhood immunization [29]. In a population with a significant reduction in PCV13 serotypes, the preventative potential of PCV13 is lower than in a population with a high proportion of circulating PCV13 serotypes. 

### 4.3. Evidence Base for PPV23

The VE of the PPV23 vaccine in our review ranged from 3% to 16% against all-cause pneumonia (Table 2). This overlaps with results from a systematic review by Kraicer-Melamed et al., where the authors found a small but non-significant protective VE of 17% in cohort studies and 7% in case–control studies [5]. Similarly, Tin Tin Htar et al. found a non-significant VE for any-CAP hospitalization of 10% in observational studies [7]. 

The VE against pneumococcal pneumonia in our review (Table 2) also overlaps with results from some previous systematic reviews. Falkenhorst reported a VE of 48% in cohort studies, 53% in a case–control study and 37 in a TND study [4], Kraicer-Melamed et al. reported a range between 5% and 45% for cohort studies and 48% in one case–control study [5], while Tin Tin Htar reported a VE of 45–53% in observational studies [7].

No other systematic reviews have reported on the VE on VT-pneumonia. In our review we found two recent studies on this topic, a TND study from Japan [21], and a case–control study from South Korea [25]. Both studies evaluated VE of PPV23 in an elderly Asiatic population.

The TND study from Japan found a VE of 34% against VT-pneumonia [21]. This study used a range of different diagnostic tools. Combining different tests can increase the overall sensitivity but decrease the overall specificity. A modeling study suggests that a decreased specificity might underestimate the true VE in observational studies [30]. Thus, one could consider the estimated VE in the Japanese study a minimum number. Sputum samples identified 208 of the 419 pneumococcal positive laboratory samples. Some of these patients might just be carriers, and this might overestimate the number of positive pneumococcal infections. In that respect, it is noteworthy that sensitivity analyses with different cut-off values for the DNA load found similar results even with a 100-fold increase. The authors found reduced VE of PPV23 by time since vaccination, with only the first two years since vaccination having a statistically significant VE (Table S3). The UK IPD surveillance study also reported a lower VE point estimate for PPV23 as the time since vaccination increased [22]. However, although the point estimate decreased from 41% for those vaccinated within two years to 23% for those vaccinated more than five years ago, the VE against VT-IPD remained statistically significant also after five years (Table S3). 

The South Korean case–control study did not find significant VE against NBPP or IPD for all age groups 65 years or older [25]. However, when stratifying by age, the authors found significant protection in patients aged 65–74 years against both outcomes (Table 3), indicating an age dependent PPV23 VE. Fifty-eight percent of the total included population was 75 years or older, and this could have affected the overall results for all patients aged 65 or older. This age dependent VE of the PPV23 was also noted in the Japanese TND study [21] and Spanish case–control study [23] (Table 3). Similarly, CAPITA did not find a significantly protective VE of PCV13 against the first episode of VT CAP in the study population aged 75 years or older (VE 32% (95% CI: -22 to 63)) [2]. The South Korean study was the only study that restricted their pneumonia cases to non-bacteremic cases. 

The VE on IPD in the studies included in our review also overlaps with some previous reviews on observational studies. Both Falkenhorst [4] and Kraicer-Melamed et al. [5] reported an estimated VE of around 50% with a 95% CI range of 15 to 74.

### 4.4. Future Perspectives

In our opinion, future studies on VE of pneumococcal vaccines should include serotype-specific information. In combination with local serotype distribution data on disease, this information can help medical and public health professionals tailor vaccination strategies against pneumococcal disease. However, in order to achieve this VE data, readily available serotype-specific diagnostic tools for non-bacteremic disease are needed. Although the SSUAD developed by Pfizer is an example of this, it can only identify serotypes included in PCV13, and it is only available for research purposes. 

## 5. Conclusions

There are strengths and weaknesses of all the included studies. We chose to include a wide range of outcomes and study designs as all of these outcomes are interesting from a public health perspective, and may add evidence in combination with earlier studies not included in this review. New high-quality observational studies indicate protective VE for both the PPV23 [21] and the PCV13 [18] against VT pneumonia. The results from those studies have overlapping CI with the results from CAPITA [2]. Our estimates for the protective VE of PPV23 on pneumonia and pneumococcal pneumonia overlap with results from previously published reviews [3,4,5,6,7]. Some of the results indicate that the VE on the PPV23 is best in younger age groups, and that it decreases over time. 

## Figures and Tables

**Table 1:** Characteristics of included studies. Y: years. PCV13: 13-valent pneumococcal conjugate vaccine. PPV23: 23-valent pneumococcal polysaccharide vaccine. RCT: randomized controlled trial. TND: Test-negative design. IPD: invasive pneumococcal disease. Pn: Pneumococcal. VT: Vaccine-type.

Author & Publication Year (Ref)	Type of Study	Country	Study Period	Time between Outcome and Vaccination	Outcome	Patient Group	Age (y)	Quality
**PCV13**								
Gessner 2018 (11)	Post hoc of RCT	Netherlands	2008-2013	Up to 5 years	Pneumonia, pn-pneumonia, VT-pneumonia, IPD and VT-IPD	Hospitalized and out-patient	≥65	NA
Huijts 2017 (12)								
Suaya 2018 (13)								
Webber 2017 (14)								
Patterson 2016 (15)								
Vila-Corcoles 2018 (16)	Cohort	Spain	2015	Not stated	Pneumonia and pn-pneumonia	Hospitalized	≥50	High
Kolditz 2018 (17)	Cohort	Germany	2014-2016	Up to 5 years	Pneumonia	Hospitalized and out-patient	≥60	High
McLaughlin 2018 (18)	TND	US	2015-2016	Up to 5 years	VT-pneumonia	Hospitalized	≥65	High
Prato 2018 (19)	TND	Italy	2013-2015	Not stated	Pn-pneumonia and VT-pneumonia	Hospitalized and out-patient	≥65	Low
**PPV23**								
Kolditz 2018 (20)	Cohort	Germany	2010-2011	Up to 5 years	Pneumonia	Hospitalized and out-patient	≥60	Low
Suzuki 2017 (21)	TND	Japan	2011-2014	Up to 5 years	Pn-pneumonia and VT-pneumonia	Hospitalized and out-patient	≥65	High
Djennad 2018 (22)	Indirect cohort	UK	2000-2016	Vaccine given at any time	VT-IPD	Hospitalized	≥65	High
Dominguez 2017 (23)	Case-control	Spain	2013-2015	Up to 5 years	Pneumonia	Hospitalized	≥65	Low
Kondo 2018 (24)	Case-control	Japan	2009-2014	Up to 5 years	Pneumonia	Outpatients	≥65	Low
Kim 2019 (25)	Case-control	South Korea	2013-2015	Up to 5 years	Pn-pneumonia ^a^, VT-pneumonia ^a^, IPD and VT-IPD	Hospitalized	≥65	High

^a^ non-bacteremic.

**Table 2:** Vaccine effectiveness (VE) percentage with 95% confidence interval by study, vaccine type and outcome. PCV13: 13-valent pneumococcal conjugate vaccine. PPV23: 23-valent pneumococcal polysaccharide vaccine. PY: Person years. Pn: pneumococcal. VT: vaccine-type. IPD: invasive pneumococcal disease.

Author (Ref)	Episodes *or* Cases Vaccinated	Individuals Vaccinated *or* Cases not Vaccinated	Episodes *or* Controls Vaccinated	Individuals not Vaccinated *or* Controls not Vaccinated	VE % Pneumonia	VE % Pn-Pneumonia	VE % VT-Pneumonia	VE % IPD	VE % VT-IPD
**PCV 13**									
Vila-Corcoles (16)	228	6912 (PY)	12471	1983789 (PY)	−69 (−94 to −48)				
	20	6912 (PY)	1628	1983789 (PY)		−17 (−83 to 25)			
Kolditz (17)	532	11395	1812	34185	11 (3 to 19)				
McLaughlin (18)	3	65	285	1681			71 (6 to 91)		
Prato (19)	5	54	15	108		33 (−107 to 82)			
	3	36	17	126			38 (−132 to 89)		
**PPV 23**									
Kolditz (20)	7501	213431	23243	640293	3 (1 to 6)				
Suzuki (21)	95	214	427	745		27 (3 to 46)			
	58	146	427	745			34 (6 to 53)		
Djennad (22)	2741	1682	1288	534					27 (17 to 35)
Dominguez (23)	259	1636	272	1623	15 (−3 to 30)				
Kondo (24)	64	170	131	307	16 (−30 to 46)				
Kim (25)	231	326	247	310		10 (−15 to 30)			
	106	137	247	310			2 (−39 to 26)		
	54	94	130	165				29 (−6 to 52)	
	21	43	130	165					42 (−2 to 67)

**Table 3:** Vaccine effectiveness (VE) percentage with 95% confidence interval by vaccine and age group. VE: Vaccine effectiveness. PPV23: 23-valent polysaccharide vaccine. PCV13: 13-valent conjugate vaccine. Pn: pneumococcal. VT: vaccine-type. IPD: invasive pneumococcal disease.

Author (Ref)	Outcome	Number of Cases ^§^	All	50–59	60–64	65–69	70–74	75–79	80–84	≥85
**PCV 13**										
Vila-Corcoles (16)	Pneumonia	12699	−69 (−94 to −48)	−21 (−72 to 15)		−76 (−104 to −52) ^A^				
	Pn-Pneumonia	1648	−17 (−83 to 25)	42 (−67 to 80)		−32 (−118 to 19)				
Kolditz (17)	Pneumonia	2344	11 (3 to 19)	−	8 (−6 to 19)				10 (−3 to 28) ^B^	
**PPV 23**										
Kolditz (20)	Pneumonia	30744	3 (1 to 6)	-	2 (−6 to 2)				0 (−4 to 3) ^C^	
Suzuki (21)	Pn-Pneumonia	419	27 (3 to 46)	-		32 (−21 to 62)		24 (−6 to 46) ^D^		
	VT-Pneumonia	272	34 (6 to 53)	-		40 (−6 to 69)		28 (−10 to 53) ^D^		
Djennad (22)	VT-IPD	4423	27 (17 to 35)	-		31 (16 to 44) ^E^		17 (−3 to 32) ^F^		34 (17 to 47) ^G^
Dominguez (23)	Pneumonia	1895	15 (−3 to 30)	-		24 (−3 to 43) ^H^		12 (−22 to 36) ^I^		0 (−58 to 37) ^J^
Kim (25)	Pn-Pneumonia	557	10 (−15 to 30)	-		35 (2 to 57)		−13 (−56 to 18) ^K^		
	VT-Pneumonia	243	−2 (−40 to 26)	-		21 (−31 to 52)		−35 (−107 to 12)		
	IPD	148	29 (−6 to 52)	-		57 (19 to 78)		7 (−74 to 50)		
	VT-IPD	64	42 (−2 to 67)	-		70 (25 to 88)		−20 (−184 to 60)		

^§^ Number of cases with outcome. ^A^ 50% (of total included participants). ^B^ 41%. ^C^ 35%. ^D^ 78%. ^E^ 38%. ^F^ 37%. ^G^ 25%. ^H^ 31%. ^I^ 47%. ^J^ 22%. ^K^ 58%.

## Supplementary Materials

The following are available online at https://www.mdpi.com/2076-0817/9/4/259/s1, Table S1: Overview of recent meta analyses of PPV23 efficacy/effectiveness. Table S2: Quality of included studies according to Newcastle–Ottawa scale and threshold categorized from Agency for Healthcare Research and Quality (AHRQ). Table S3: Vaccine efficacy/effectiveness (VE) percentage including 95% confidence interval of the 23-valent polysaccharide vaccine by time since vaccination. Pn: pneumococcal. VT: vaccine-type. IPD: invasive pneumococcal disease. Appendix A: Search strategy. Appendix B: data extraction form. Appendix C: PICO.

## Abbreviations

CAP: Community acquired pneumonia. IPD: invasive pneumococcal disease. NBPP: non-bacteremic pneumococcal pneumonia. NOS: Newcastle–Ottawa Scale. PCV13: 13-valent conjugate vaccine. Pn: pneumococcal. PPV23: 23-valent polysaccharide vaccine. RCT: randomized controlled trials. TND: test-negative design. VE: Vaccine efficacy (clinical trials) or vaccine effectiveness (observational studies). VT: vaccine-type.
