# Supplementary material for: A Systematic Review of Studies Published between 2016 and 2019 on the Effectiveness and Efficacy of Pneumococcal Vaccination on Pneumonia and Invasive Pneumococcal Disease in an Elderly Population
Source: Pathogens. 2020 Apr 3;9(4):259. doi: 10.3390/pathogens9040259 (PMC7238108; doi:10.3390/pathogens9040259)
Supplement: Supplementary file 1 [file pathogens-09-00259-s001.pdf]

pathogens

Table S1. Overview of recent meta analyses of PPV23 efficacy/effectiveness. Modified from table 3 in Falkenhorst et al [4]. RCT: Randomized controlled trial. Obs: Observational studies. NR: Not reported.

**Author, Year**	**Inclusion Criteria**		**Pooled Vaccine Efficacy/Effectiveness in % Including (95% Confidence Interval)**		
	**Age Group (years)**	**Study Design**	**IPD, any serotype**	**Pneumococcal Pneumonia, Any Serotype**	**All-cause CAP**
Kraicer-Melamed 2016	50+	RCT	NR	range -28 (-90 to 20) to 22 (-51 to 60)[1]	-10 (-36 to 12)
		Cohort	50 (21 to 69)	Range 5 to 45[1]	17 (-26 to 45)
		Case-control	54 (32 to 69)	48 (27 to 63)[2]	7 (-10 to 21)
Diao, 2016	15+	RCT	NR	46 (-65 to 82)	13 (2 to 24)
		Obs.		-	
Schiffner-Rohe, 2016	65+	RCT	NR	-11 (-93 to 36)	-10 (-30 to 7)
		Obs.		-	
Falkenhorst, 2017	60+	RCT	73 (10 to 92)	64 (35 to 80)	NR
		Cohort	45 (15 to 65)	48 (25 to 63)	NR
		Case-control	59 (35 to 74)	53 (33 to 68) [2]	
Tin Tin Htar, 2017	16+	RCT		-	
		Obs.	NR	Range 45 (12 to 66) to 53 (33 to 68) [1]	5 (-2 to 12)

[1]No pooled estimate reported; [2]Only one study included.

Table S2. Quality of included studies according to Newcastle-Ottawa scale and threshold categorized from Agency for Healthcare Research and Quality (AHRQ).

**Case Control, Test-Negative Design and Indirect Cohort Studies**						
Author (ref)	Vaccine	Selection (x/4)	Comparability[#] (x/2)	Exposure (x/3)	Total (x/9)	Quality[¤]
McLaughlin (18)	PCV13	4	2	3	9	High
Prato (19)	PCV13	2	0	3	5	Low
Suzuki (21)	PPV23	4	2	3	9	High
Djennad (22)	PPV23	4	2	2	8	High
Dominguez (23)	PPV23	2	2	1	5	Low
Kondo (24)	PPV23	2	2	1	5	Low
Kim (25)	PPV23	3	2	3	8	High
**Cohort studies**						
Author (ref)	Vaccine	Selection (x/4)	Comparability[#] (x/2)	Outcome (x/3)	Total (x/9)	
Vila-Corcoles (16)	PCV13	3	2	2	7	High
Kolditz (17)	PCV13	4	2	2	8	High
Kolditz (20)	PPV23	4	2	1	7	Low

Case control, test-negative design and indirect cohort studies: *Selection*; is the case definition adequate, representativeness of the cases, selection of controls, definition of controls, *Comparability*; study controls for most important factors, *Exposure*; ascertainment of exposure, same method of ascertainment for cases and controls, non-response rate.

Cohort studies: *Selection;* Representativeness of the exposed cohort, selection of the non-exposed cohort, ascertainment of exposure, demonstration of outcome not present at enrolment, *Comparability*; study controls for most important factors, *Outcome*; assessment of outcome, was follow-up long enough for outcomes to occur, adequacy of follow-up of cohorts.

[#]On comparability, studies earned one star if they adjusted for age and comorbidities, and two stars if adjustment also included separate adjustments for immunocompetent and immunosuppressive conditions. .

[¤]We applied following thresholds for converting NOS to categorized standards from Agency for Healthcare Research and Quality:.

- High: 3 or 4 stars in selection domain AND 1 or 2 stars in comparability domain AND 2 or 3 stars in outcome/exposure domain
- Moderate: 2 stars in selection domain AND 1 or 2 stars in comparability domain AND 2 or 3 stars in outcome/exposure domain
- Low: 0 or 1 star in selection domain OR 0 stars in comparability domain OR 0 or 1 stars in outcome/exposure domain

Table S3. Vaccine efficacy/effectiveness (VE) percentage including 95% confidence interval of the 23-valent polysaccharide vaccine by time since vaccination. Pn: pneumococcal. VT: vaccine-type. IPD: invasive pneumococcal disease.

Author (ref)	Outcome	VE by Time Since Vaccination (years)					
		**0-1**	**>1-2**	**>2-3**	**>3-4**	**>4-5**	**>5**
Kolditz (20)	pneumonia	-5.5(2.2 to -13.3)	-2.8(3.8 to -9.5)	-6.9(-0.7 to -12.9)	10.3(14.4 to 5.3)	5.2(9.7 to 0.2)	NA
		**0 to <2**		**2 to <5**			**>5**
Suzuki (21)	Pn-Pneumonia	31.5 (1.5 to 51.0)		25.7 (-11.5 to 50.5)			0.2 (-76.5 to 27.3)
	VT-Pneumonia	37.7 (4.8 to 59.2)		34.7 (-7.4 to 60.3)			26 (-56.4 to 65.0)
Djennad (22)	VT-IPD	41 (23 to 54)		34 (16 to 48)			23 (12 to 32)

Appendix A: Search strategy.

Database: Ovid MEDLINE(R) and Epub Ahead of Print, In-Process & Other Non-Indexed Citations, Daily and Versions(R) 1946 to date of search.

1	streptococcus pneumoniae/
2	(pneumococcal or pneumococci or "streptococcus pneumonie" or "streptococcus pneumoniae" or "s pneumoniae").tw,kf.
3	1 or 2
4	vaccination/ or immunization/ or Vaccines, Conjugate/
5	(vaccine? or vaccination? or immunisation or immunization).tw,kf.
6	4 or 5
7	Pneumococcal Vaccines/
8	(heptavalent or "7 valent" or "7valent" or "pcv 7" or pcv7* or "10 valent" or "10valent" or pcv10 or "pcv 10" or "13valent" or "13 valent" or pcv13 or "pcv 13" or ppv23 or "ppv 23" or "23 valent" or "23valent").tw,kf.
9	7 or 8
10	(3 and 6) or 9
11	comparative effectiveness research/ or treatment outcome/ or Vaccine Potency/
12	(efficacy or effectiveness or effects or "immune response" or impact or "treatment outcome").tw,kf.
13	11 or 12
14	adult/ or aged/ or "aged, 80 and over"/ or frail elderly/
15	(adult? or elderly or elderlies).tw,kf.
16	14 or 15
17	10 and 13 and 16
18	2019*.ed,ep,yr,dp,dc.
19	(201808* or 201809* or 201810* or 201811* or 201812*).ep,ed,dc.
20	18 or 19
21	17 and 20

Database: Embase 1974 to date of search.

1	Streptococcus pneumoniae/
2	(pneumococcal or pneumococci or "streptococcus pneumonie" or "streptococcus pneumoniae" or "s pneumoniae").tw,kw.

3	1 or 2
4	vaccination/ or immunization/ or vaccine/
5	(vaccine? or vaccination? or immunisation or immunization).tw,kw.
6	4 or 5
7	Pneumococcus vaccine/
8	(heptavalent or "7 valent" or "7valent" or "pcv 7" or pcv7* or "10 valent" or "10valent" or pcv10 or "pcv 10" or "13valent" or "13 valent" or pcv13 or "pcv 13" or ppv23 or "ppv 23" or "23 valent" or "23valent").tw,kw.
9	7 or 8
10	(3 and 6) or 9
11	comparative effectiveness/ or treatment outcome/
12	(efficacy or effectiveness or effects or "immune response" or impact or "treatment outcome").tw,kw.
13	11 or 12
14	aged/ or adult/ or aged hospital patient/ or frail elderly/ or institutionalized elderly/ or very elderly/
15	(adult? or elderly or elderlies).tw,kw.
16	14 or 15
17	10 and 13 and 16
18	limit 17 to yr="2016 -Current"
19	limit 18 to (conference abstracts or embase)

Database: Cinahl.

S17	S9 AND S12 AND S15 Limiters - Published Date: 20160101-20191231; Exclude MEDLINE records
S16	S9 AND S12 AND S15
S15	S13 OR S14
S14	TI (adult# or elderly or elderlies) OR AB (adult# or elderly or elderlies)
S13	(MH "Adult") OR (MH "Aged+")
S12	S10 OR S11
S11	TI (efficacy or effectiveness or effects or "immune response" or impact or "treatment outcome") OR AB (efficacy or effectiveness or effects or "immune response" or impact or "treatment outcome")

S10	(MH "Treatment Outcomes")
S9	S7 OR S8
S8	S1 AND S4
S7	S5 OR S6
S6	TI (heptavalent or "7 valent" or "7valent" or "pcv 7" or pcv7* or "10 valent" or "10valent" or pcv10 or "pcv 10" or "13valent" or "13 valent" or pcv13 or "pcv 13" or ppv23 or "ppv 23" or "23 valent" or "23valent") OR AB (heptavalent or "7 valent" or "7valent" or "pcv 7" or pcv7* or "10 valent" or "10valent" or pcv10 or "pcv 10" or "13valent" or "13 valent" or pcv13 or "pcv 13" or ppv23 or "ppv 23" or "23 valent" or "23valent")
S5	(MH "Pneumococcal Vaccine")
S4	S2 OR S3
S3	TI (vaccine# or vaccination# or immunisation or immunization) OR AB (vaccine# or vaccination# or immunisation or immunization)
S2	(MH "Vaccines") OR (MH "Immunization")
S1	TI (pneumococcal or pneumococci or "streptococcus pneumonie" or "streptococcus pneumoniae" or "s pneumoniae") OR AB (pneumococcal or pneumococci or "streptococcus pneumonie" or "streptococcus pneumoniae" or "s pneumoniae")

Database: Web of Science.

# 9	#7 AND #6 AND #5<br>*Indexes=SCI-EXPANDED, SSCI, A&HCI, ESCI Timespan=2016-2019*
# 8	#7 AND #6 AND #5<br>*Indexes=SCI-EXPANDED, SSCI, A&HCI, ESCI Timespan=All years*
# 7	**TOPIC:** (("adult$" or "elderly" or "elderlies"))<br>*Indexes=SCI-EXPANDED, SSCI, A&HCI, ESCI Timespan=All years*
# 6	**TOPIC:** (("efficacy" or "effectiveness" or "effects" or "immune response" or "impact" or "treatment outcome"))<br>*Indexes=SCI-EXPANDED, SSCI, A&HCI, ESCI Timespan=All years*
# 5	#4 OR #3<br>*Indexes=SCI-EXPANDED, SSCI, A&HCI, ESCI Timespan=All years*
# 4	**TOPIC:** (("heptavalent" or "7 valent" or "7valent" or "pcv 7" or pcv7* or "10 valent" or "10valent" or "pcv10" or "pcv 10" or "13valent" or "13 valent" or "pcv13" or "pcv 13" or "ppv23" or "ppv 23" or "23 valent" or "23valent"))

	Indexes=SCI-EXPANDED, SSCI, A&HCI, ESCI Timespan=All years
# 3	#2 AND #1<br>*Indexes=SCI-EXPANDED, SSCI, A&HCI, ESCI Timespan=All years*
# 2	**TOPIC:** (("vaccine$" or "vaccination$" or "immunisation" or "immunization"))<br>*Indexes=SCI-EXPANDED, SSCI, A&HCI, ESCI Timespan=All years*
# 1	**TOPIC:** (("pneumococcal" or "pneumococci" or "streptococcus pneumonie" or "streptococcus pneumoniae" or "s pneumoniae"))<br>*Indexes=SCI-EXPANDED, SSCI, A&HCI, ESCI Timespan=All years*

Database: Epistemonikos.

Search 1:

(pneumococcal OR pneumococci OR "streptococcus pneumonie" OR "streptococcus pneumoniae" OR "s pneumoniae") AND (vaccine* OR vaccination* OR immunisation OR immunization) AND (efficacy OR effectiveness OR effects OR "immune response" OR impact OR "treatment outcome") AND (adult* OR elderly OR elderlies) – limited to 2016-2019

= 8 hits

Search 2:

(heptavalent OR "7 valent" OR "7valent" OR "pcv 7" OR pcv7* OR "10 valent" OR "10valent" OR pcv10 OR "pcv 10" OR "13valent" OR "13 valent" OR pcv13 OR "pcv 13" OR ppv23 OR "ppv 23" OR "23 valent" OR "23valent") AND (efficacy OR effectiveness OR effects OR "immune response" OR impact OR "treatment outcome") AND (adult* OR elderly OR elderlies) – limited to 2016-2019

= 5 hits

Appendix B: data extraction form.

Author
Year of publication
Source of publication
Journal
Study design
Country
Population
Age
Study Period
Total participants at entry/eligible
Total participants analysis

Selection of controls
Exclusion criteria
Childhood vaccination program
Intervention
Details vaccination
Comparator
Outcome
IPD
IPD_VT
All cause pneumonia
Hospitalized
pnPneumonia
VT_Pneumonia
Other_Pneumonia
Age stratified outcomes
Outcome stratified by immunstatus
Outcome stratified by other factor (specify)
Defintion CAP
Other diagnostic tools
Episodes(Cases)/N intervention group
Episodes(Control)/N comparator group
N not in alanlysis
Years of follow-up/time since vaccination

Appendix C: PICO

Population: Elderly population

Intervention: PPV23 or PCV13

Comparator: Placebo or no vaccine

Outcome: IPD and/or pneumonia
